# A preliminary investigation on the possible association between diminished copper availability and non-alcoholic fatty liver disease in epileptic patients treated with valproic acid

**DOI:** 10.3109/03009734.2010.545898

**Published:** 2011-04-12

**Authors:** Natalia Lampon, J. Carlos Tutor

**Affiliations:** Unidad Monitorización Fármacos, Laboratorio Central, Hospital Clínico Universitario, Instituto de Investigación Sanitaria (IDIS), Santiago de Compostela, Spain

**Keywords:** APRI, butyrylcholinesterase, copper deficiency, FIB-4, non-alcoholic fatty liver disease, valproic acid

## Abstract

**Background:**

Patients treated with valproic acid (VPA) present a high incidence of non-alcoholic fatty liver disease (NAFLD) (around 61%). Several recent studies suggest that low copper stores could be associated with NAFLD, and a significant decrease of copper availability in VPA-treated patients has been described.

**Design and methods:**

In 101 adult epileptic patients treated with valproic acid in monotherapy (*n* = 75) and polytherapy (*n* = 26) the copper availability was evaluated using the specific oxidase activity of ceruloplasmin (activity per unit mass of enzyme protein) and the copper/ceruloplasmin ratio. Copper deficiency was supposed in the cases in which this biochemical variable was smaller than the lower reference limit (333 U/g).

**Results:**

The differences between the groups of patients with ceruloplasmin oxidase activity smaller or greater than 333 U/g for the serum levels of aminotransferases, gamma-glutamyltransferase, butyrylcholinesterase, cholesterol, triglycerides, and C-reactive protein, and the APRI and FIB-4 liver fibrosis scores were not statistically significant. Most patients (93%) had low APRI and FIB-4 scores, suggesting absence of significant liver fibrosis.

**Conclusions:**

The results obtained do not confirm the hypothesis of an association between diminished copper availability and NAFLD in patients treated with valproic acid.

## Introduction

Valproic acid (VPA) has been widely used since the late 1960s for the treatment of generalized and partial seizures, and it is still the antiepileptic drug with the broadest spectrum (1). New roles for this old drug were later confirmed, due to its beneficial effects in bipolar disorders, schizophrenia, depression, neurological pain, migraine headaches, and a number of neurodegenerative diseases (2,3).

It has been highlighted that on rare occasions serious complications may occur in some VPA-treated patients, including pancreatitis, coagulopathies, induced hepatotoxicity, and encephalopathy, although there is still a lack of knowledge about the incidence of these special side effects (1). Type I VPA-associated hepatotoxicity consists of a dose-dependent elevation of serum liver enzymes, which normalize after drug discontinuation, and type II consists of a rare idiosyncratic VPA-associated hepatotoxicity that is normally lethal (1).

VPA has a significant steatogenic effect in HpG2 cell cultures (4), and development of fatty liver is one of the clinical findings during treatment with this drug (5). Using abdominal ultrasound, Luef et al. demonstrated that non-alcoholic fatty liver disease was present in 61% of VPA-treated patients (6), with this high prevalence being confirmed in a more recent study (7). Impaired hepatic fatty acid oxidation directly inhibiting mitochondrial β-oxidation enzymes, or sequestering co-factors involved in this metabolic pathway, has been proposed as a possible mechanism for VPA-induced steatosis(4,5,8).

Chronic treatment with enzyme-inducing antiepileptic drugs (carbamazepine, phenobarbital, or phenytoin) does not produce copper deficiency (9). However, the administration of VPA produces a significant increase of copper biliary excretion in rats (10), and previously published results suggest that diminished copper availability is substantially prevalent among epileptic patients treated with VPA (11). In accordance with Aigner et al., a significant proportion of non-alcoholic fatty liver disease patients should be considered copper-deficient (12), and reduced copper availability may be involved in the development of non-alcoholic fatty liver (13), a hypothesis put forward at an earlier date by Geubel et al. (14).

In our study, an investigation was carried out in epileptic patients treated with VPA on the possible relationship between copper availability and the serum activities of several liver enzymes, whose increase in non-alcoholic fatty liver disease is widely documented (15–22). As previously reported, copper deficiency was supposed in cases with a smaller ceruloplasmin-specific oxidase activity (activity per mass unit of enzyme protein) than the estimated lower limit of reference (11).

## Patients and methods

A group of 101 epileptic out-patients (56 males and 45 females) with a mean age (±SEM) of 43.7 ± 2.5 years (range 17–84 years) and treated with VPA in monotherapy (*n* = 75) or polytherapy with associated phenytoin, carbamazepine, and phenobarbital (*n* = 26) were studied. In all cases the daily drug administration was carried out in multiple doses. The blood samples were drawn before the breakfast and the morning dose of antiepileptic drugs, which had not been modified for at least 3 months prior to the study. Consequently the serum levels of VPA correspond to the trough steady-state concentrations (Css). None of the patients was receiving a copper dietary supplement, and pregnant women or those taking oral contraceptives were excluded. This study was carried out according to the good practice rules for investigation in humans of the Conselleria de Sanidade (Regional Ministry of Health) of the Xunta de Galicia, Spain.

Serum copper concentration was determined by atomic absorption spectrometry (Perkin Elmer mod 5100PC, Waltham, USA) with carbon red atomization. Immunoreactive ceruloplasmin was determined in a BN ProSpec nephelometer (Siemens Health Care Diagnostics Inc., Newark, USA). The oxidase activity of ceruloplasmin was determined as previously described at 30°C using o-dianisidine dihydrochloride as a substrate (23), and the ceruloplasmin-specific oxidase activity was calculated using the expression: specific oxidase activity (U/g) = 1000 × oxidase activity (U/L)/immunoreactive ceruloplasmin (mg/L). Serum VPA concentrations were determined in a Dimension Xpand analyzer using reagents from Siemens Health Care Inc. Serum activities of alanine aminotransferase (ALT), aspartate aminotransferase (AST), gamma-glutamyltransferase (GGT), alkaline phosphatase (ALP),and butyrylcholinesterase (ChE), and concentrations of glucose, cholesterol, and triglycerides, were determined in an Advia 2400 Chemistry System (Siemens Health Care Diagnostics Inc.). The platelet count, mean platelet volume (MPV), and platelet size distribution width (PDW) were measured in blood samples collected 2–3 hours beforehand in K_3_EDTA anticoagulated tubes using an Advia 2120 Hematology System (Siemens Health Care Diagnostics Inc.). High-sensitivity C-reactive protein (CRP) was determined in a BN II nephelometer (Siemens Health Care Diagnostics Inc.).

The AST-to-platelet ratio index (APRI) was calculated in accordance with Wai et al. (24): APRI = (AST:URL/platelet count (10^9^/L)) × 100, where URL corresponds to the AST upper reference limits for men and women. The 4-variables liver fibrosis index (FIB-4) was calculated in accordance with Sterling et al. (25): FIB-4 = age × AST/platelets (10^9^/L) × ALT^1/2^, where age is expressed in years and the AST and ALT activities in U/L.

Statistical analysis of data was performed using the StatGraphics package, and the Kolmogorov-Smirnov test was applied to check for normality. Pearson's correlation coefficient was used when the data had a Gaussian distribution. Otherwise, Spearman's correlation coefficient was used. The results were expressed as mean ± SEM (median).

## Results

The estimated lower reference limit for the specific oxidase activity of ceruloplasmin (2.5 percentile of the control group) was 333 U/g (11). Table I shows the results obtained for the different biochemical variables assayed in the patients treated with VPA in monotherapy, grouped according to their ceruloplasmin-specific oxidase activities (smaller or greater than 333 U/g). The differences between the mean (median) were not statistically significant, with the exception of the data obtained for the copper/immunoreactive ceruloplasmin ratio, which was significantly lower in the group of patients with ceruloplasmin-specific oxidase activities <333 U/g. Analogous results were obtained for the patients treated with VPA in polytherapy (Table II). In both cases, significant differences were not achieved after a dichotomy of the data according to the patient's sex. No significant correlations were obtained for the different variables assayed with the specific oxidase activity of ceruloplasmin or the copper/immunoreactive ceruloplasmin ratio, which have a significant correlation coefficient between them (*r* = 0.550; *P* < 0.001).

**Table I.** Results obtained in the patients treated with valproic acid in monotherapy according to its ceruloplasmin-specific oxidase activity.

	Specific oxidase activity <333(U/g)	Specific oxidase activity ≥333(U/g)
*n* (males/females)	28 (18/10)	47 (21/26)
VPA Css (mg/L)	63.2 ± 4.0 (67.9)	63.5 ± 2.7 (60.4)
VPA dose (mg/24h)	1170.0 ± 93.1 (1200.0)	1241.9 ± 86.1 (1000.0)
Specific oxidase activity (U/g)	278.5 ± 6.8 (276.1)^a^	430.5 ± 13.0 (405.4)
Copper/ceruloplasmin ratio (μg/mg)	3.2 ± 0.1 (3.2)^a^	3.9 ± 0.1 (3.8)
Glucose (mg/dL)	94.5 ± 5.1 (89.0)	88.4 ± 2.0 (87.5)
Cholesterol (mg/dL)	191.0 ± 10.2 (199.0)	175.5 ± 8.2 (171.0)
Triglycerides (mg/dL)	134.5 ± 13.2 (108.0)	107.7 ± 8.9 (99.0)
GGT (U/L)	31.6 ± 8.8 (12.6)	17.2 ± 2.5 (10.0)
ALP (U/L)	161.2 ± 11.3 (132.0)	162.1 ± 3.7 (143.0)
ChE (U/L)	7061.6 ± 407.4 (7250.0)	7005.9 ± 229.4 (7050.0)
AST(U/L)	14.6 ± 1.1 (13.5)	16.7 ± 1.9 (13.0)
ALT(U/L)	19.2 ± 1.6 (20.0)	22.1 ± 3.9 (14.5)
AST/ALT ratio	0.8 ± 0.1 (0.8)	0.9 ± 0.1 (0.7)
CRP (mg/L)	3.4 ± 1.2 (1.4)	6.6 ± 2.6 (1.4)
APRI score	0.37 ± 0.06(0.30)	0.35 ± 0.05(0.30)
FIB-4 score	0.89 ± 0.12(0.80)	0.84 ± 0.12(0.60)

^a^Significance: *P* < 0.01.

**Table II.** Results obtained in the patients treated with valproic acid in polytherapy according to its ceruloplasmin-specific oxidase activity.

	Specific oxidase activity<333(U/g)	Specific oxidase activity ≥333(U/g)
*n* (males/females)	10 (7/3)	16 (10/6)
VPA Css (mg/L)	49.4 ± 4.1 (48.1)	49.1 ± 6.3 (41.5)
VPA dose (mg/24h)	1900.0 ± 362.5 (1500.0)	1733.3 ± 244.5 (1500.0)
Specific oxidase activity (U/g)	256.0 ± 19.5 (279.8)^b^	454.3 ± 18.0 (439.3)
Copper/ceruloplasmin ratio (μg/mg)	3.3 ± 0.2 (3.2)^a^	3.9 ± 0.2 (3.9)
Glucose (mg/dL)	85.4 ± 1.7 (86.0)	98.9 ± 7.8(87.5)
Cholesterol (mg/dL)	215.4 ± 19.6 (194.0)	222.8 ± 23.5 (209.0)
Triglycerides (mg/dL)	126.8 ± 17.6 (130.5)	130.2 ± 25.0 (102.0)
GGT (U/L)	65.0 ± 19.3 (50.5)	70.6 ± 18.2 (44.0)
ALP (U/L)	145.2 ± 21.1 (147.0)	201.2 ± 34.1 (164.5)
ChE (U/L)	7198.6 ± 845.9 (7308.0)	7638.0 ± 798.0 (7147.0)
AST(U/L)	17.6 ± 4.4 (14.5)	15.9 ± 1.7 (15.0)
ALT(U/L)	27.4 ± 9.2 (19.0)	21.1 ± 3.1 (16.0)
AST/ALT ratio	0.8 ± 0.1 (0.7)	0.9 ± 0.1 (0.8)
CRP (mg/L)	38.7 ± 26.0 (8.9)	27.0 ± 14.7 (4.9)
APRI score	0.33 ± 0.07(0.30)	0.25 ± 0.04 (0.20)
FIB-4 score	0.69 ± 0.12(0.70)	0.63 ± 0.15 (0.50)

^a^Significance: *P* < 0.05.

^b^Significance: *P* < 0.001.

In our study, thrombocytopenia was defined as a platelet count of less than 150 × 10^9^/L, and 11 patients (10 in monotherapy and 1 in polytherapy ) had thrombocytopenia, with a platelet count of 115 ± 9.7 × 10^9^/L (range 42–146 × 10^9^/L), MPV of 9.5 ± 0.4 fL (range 7.6–11.5 fL), and PDW of 54.8 ± 2.4% (range 39.8%–63.8%). In accordance with previously published data on the discrimination of hypo-productive or hyper-destructive thrombocytopenia using MPV and PDW platelet indices (26,27), the decreased platelet count may be due to a hyper-destruction of peripheral blood platelets in these patients. Furthermore, in the five patients (three in monotherapy and two in polytherapy) with a platelet count greater than 400 × 10^9^/L, the calculation of the residual PDW according to Osselaer et al. (28) suggests that the discrete thrombocytosis of 491.8 ± 37.7 × 10^9^/L (range 409–601 × 10^9^/L) was secondary or reactive. No significant correlations of the platelet count with the daily dose or serum levels of VPA were found.

**Figure 1.**
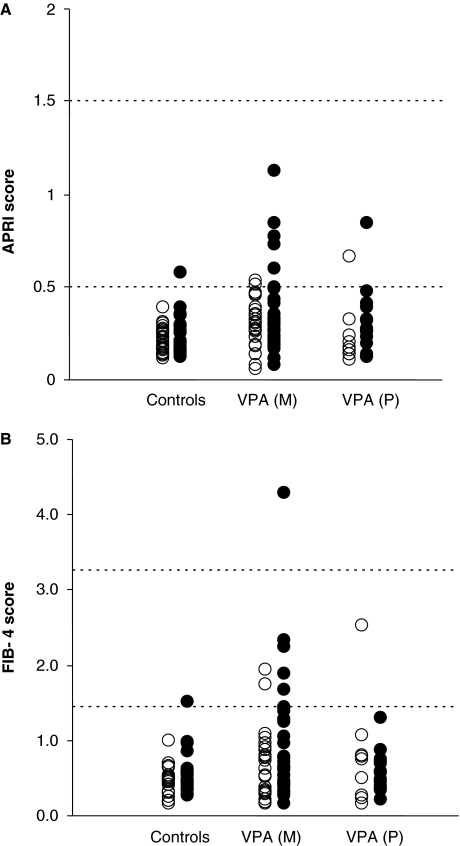
Distribution of the APRI score (A) and FIB-4 score (B) in males (•) and females (○) controls and patients treated with valproic acid in monotherapy (VPAM) and polytherapy (VPAP). The dashed lines correspond to the cut-off values for absence (APRI <0.5; FIB-4 <1.45) or presence of significant liver fibrosis (APRI>1.5; FIB-4 >3.25).

Figure 1 shows the distribution of the values obtained for the APRI and FIB-4 liver fibrosis indices in 50 healthy controls and the patients who were studied. The correlation coefficient of the APRI index with the platelet count was *r* = –0.764 (*P* < 0.001), and with AST activity *r* = 0.635 (*P* < 0.001). The correlation of FIB-4 score with platelet count (*r* = –0.606;*P* < 0.001) was also greater than with AST (*r* = 0.348;*P* < 0.005). The first-order partial correlation coefficient of the APRI index with platelet count (maintaining AST as a constant) was *r* = –0.898 (*P* < 0.001), and with AST activity (maintaining platelet count as a constant) *r* = 0.849 (*P* < 0.001). The partial correlations of the FIB-4 score with the platelet count was *r* = –0.607 (*P* < 0.001), and with AST *r* = 0.350 (*P* < 0.005).

## Discussion

It has been previously described that non-alcoholic fatty liver disease is present in 61% of the patients treated with VPA (6,7), and around 38% of these patients may have a diminished copper availability (11), a particular condition that would be involved in the development of liver steatosis (12–14). A relevant proportion of non-alcoholic fatty liver disease patients had low copper stores (12,13), which is associated with more pronounced insulin resistance and other clinical features of the metabolic syndrome, and might be a typical feature of pathophysiological relevance to its hepatic manifestation as non-alcoholic fatty liver disease (12,13). Patients with serum copper levels in the currently applied ‘normal’ range may include a significant proportion of cases with copper stores that can be considered depleted (12).

The specific oxidase activity of circulating ceruloplasmin, which in adults is not influenced by non-dietary factors such as age, gender, or hormone use, is a more sensitive indicator of copper status than either serum copper, ceruloplasmin, or erythrocyte superoxide dismutase (29,30). In the VPA-treated patients with ceruloplasmin-specific oxidase activity below the lower limit of reference (333 U/L) a reduced copper content of the circulating ceruloplasmin due to the diminished liver copper availability should be supposed (11).

Recently, it has been reported that the severity of non-alcoholic fatty liver disease correlates with high-sensitivity CRP values (31). In our patients, a significant relationship of the specific oxidase activity of ceruloplasmin with CRP levels was not attained, indicating that the specific oxidase activity is not influenced by the inflammatory status. Significant increases of the serum AST, ALT, GGT, and ChE activities in groups of patients with non-alcoholic fatty liver disease are well documented (15–22), as well as its correlation with the grade of steatosis and fibrosis assessed by liver ultrasound (17,19,20). In particular, increased serum ChE is considered a useful marker of liver steatosis (21,32), associated with adiposity, insulin resistance, lipid profile (22,33), and fatty liver infiltration degree (34). The serum levels of AST, ALT, GGT, and ChE, and AST/ALT ratio, APRI and FIB-4 indices were analogous and without significant differences between the groups of VPA-treated patients with ceruloplasmin-specific oxidase activities lower or greater than 333 U/L (Tables I and II). These results do not confirm the hypothesis of a significant association between copper deficiency and fatty liver disease in the patients treated with VPA.

It has been hypothesized that elevated ChE activities could lead to a decrease of acetylcholine which has anti-inflammatory actions and consequently could trigger the onset of low-grade systemic inflammation (35,36). Significant increases of serum ChE (37,38) and CRP (39,40) have been reported in epileptic patients treated with different anticonvulsant drugs. In our patients having CRP concentrations lower than 10 mg/L (subclinical chronic inflammation), a significant positive correlation between serum ChE and CRP was found (*r* = 0.410;*P* < 0.001); however, in the cases with CRP greater than 10 mg/L (acute inflammation conditions) the correlation coefficient between these two variables was negative, although due to the low number of patients considered, statistical significance was not achieved (*r* = –0.405;*P* = 0.084). Similarly, in the group of patients with CRP levels lower than 10 mg/L the ChE activity was significantly higher (*P* < 0.005) than in the patients with CRP greater than 10 mg/L (7,400.9 ± 189.7 U/L (7,309.0 U/L) versus 5,846.5 ± 681.2 U/L (6,004.0 U/L)), possibly due to a greater liver injury in these last patients. This is an interesting subject that should be studied in a greater detail.

Non-alcoholic fatty liver disease ranges from simple steatosis, with a benign course, to non-alcoholic steatohepatitis with necroinflammation and fibrosis, which can progress to cryptogenic cirrhosis and end-stage liver disease. As shown in Figure 1, most control subjects (98%) and VPA-treated patients (93%) have low scores for the APRI (<0.5) and FIB-4 (<1.45) indices, suggesting the absence of liver fibrosis in these cases. Likewise, in the remaining patients the values obtained for both indices suggest a low likelihood of significant hepatic fibrosis. Only a 95-year-old male patient with platelet count of 107 × 10^9^/L, AST 16 U/L, and ALT 11 U/L (AST/ALT ratio = 1.45), and presenting low serum levels of GGT 12 U/L, ChE 4,621 U/L, and albumin 31 g/L, had a FIB-4 score of 4.3 indicative of severe liver fibrosis, although the APRI index was 0.60.

The variables AST activity and platelet count are used for the calculation of the APRI (24) and FIB-4 (25) liver fibrosis indices. With the progression of liver fibrosis, serum AST activity increases due to its release from mitochondria and its diminished clearance; likewise, the platelet count decreases due to a hypo-production of thrombopoietin by the hepatocytes and an increased circulating platelet destruction in spleen, as portal hypertension develops. In the VPA-treated patients studied, the coefficient of determination (estimated from the correspondent first partial correlation coefficients indicated above) shows that the APRI score is slightly more dependent on the platelet count (*r^2^* = 0.806) than AST activity (*r^2^* = 0.721). The dependence of FIB-4 score on the platelet count (*r^2^* = 0.368) was also higher than with AST activity (*r^2^* = 0.122).

Considering the MPV and PDW platelet indices of our 11 patients with thrombocytopenia, this fact may be due to a hyper-destruction of the circulating platelets as was indicated above. Thrombocytopenia is the most common hematologic adverse effect of VPA, although the exact mechanism has not yet been elucidated (41). Our study did not reveal any significant correlation between serum VPA concentration and platelet count, or significant higher VPA levels in the 11 patients with platelet count lower than 150 × 10^9^/L with respect to the remaining patients (55.4 ± 7.7 mg/L (48.1 mg/L) versus 59.4 ± 2.5 mg/L (56.5 mg/L)), suggesting that thrombocytopenia in these cases was not mediated by direct drug toxicity on the blood platelets. However, a VPA-induced formation of autoantibodies against platelets is possible (41), and the results obtained for the MPV and PDW indices in our patients are compatible with a hyper-destruction or reduced circulating platelet survival, similar to that seen in immune thrombocytopenia. If this assumption is correct, the APRI and FIB-4 scores would be falsely increased, at least in some cases, reinforcing the suggestion expressed above that in the studied group of VPA-treated patients there is no significant liver fibrosis. With regard to these results, the recently described *in-vitro* and *in-vivo* inhibitory effect of VPA on the hepatic stellate cell activation, and consequently on fibrosis development in chronically injured mouse liver (42), should be considered.

Unfortunately a diagnosis of non-alcoholic fatty liver disease by ultrasonography was not carried out in our study. In any case, the results we have obtained suggest that the decreased copper availability present in the epileptic patients treated with VPA is not associated with fatty liver disease. Copper status is linked to iron homeostasis, and copper deficiency causes hepatic iron accumulation that is frequently associated with hepatic steatosis (12). Increased liver iron can promote oxidative stress and insulin resistance; however, these processes may be modulated by haptoglobin polymorphism, and Hp2-2 phenotype would be a risk factor for metabolic syndrome and non-alcoholic fatty liver disease (43). The association between copper availability, iron metabolism, haptoglobin polymorphism, and metabolic syndrome requires clarification.
